# Computational learning of features for automated colonic polyp classification

**DOI:** 10.1038/s41598-021-83788-8

**Published:** 2021-02-23

**Authors:** Kangkana Bora, M. K. Bhuyan, Kunio Kasugai, Saurav Mallik, Zhongming Zhao

**Affiliations:** 1 Department of Computer Science and IT, Cotton University, Pan Bazar, Guwahati, Assam 781001 India; 2 Department of Electrical and Electronics Engineering, Indian Institute of Technology Guwahati (IITG), Guwahati, Assam 781039 India; 3 Department of Gastroenterology, Aichi Medical University, Nagakute, 480-1195 Japan; 4 Center for Precision Health, School of Biomedical Informatics, The University of Texas Health Science Center at Houston, Houston, TX 77030 USA; 5 Human Genetics Center, School of Public Health, The University of Texas Health Science Center at Houston, Houston, TX USA; 6 Department of Pathology and Laboratory Medicine, McGovern Medical School, The University of Texas Health Science Center at Houston, Houston, TX USA

**Keywords:** Computational biology and bioinformatics, Diseases, Health care

## Abstract

Shape, texture, and color are critical features for assessing the degree of dysplasia in colonic polyps. A comprehensive analysis of these features is presented in this paper. Shape features are extracted using generic Fourier descriptor. The nonsubsampled contourlet transform is used as texture and color feature descriptor, with different combinations of filters. Analysis of variance (ANOVA) is applied to measure statistical significance of the contribution of different descriptors between two colonic polyps: non-neoplastic and neoplastic. Final descriptors selected after ANOVA are optimized using the fuzzy entropy-based feature ranking algorithm. Finally, classification is performed using Least Square Support Vector Machine and Multi-layer Perceptron with five-fold cross-validation to avoid overfitting. Evaluation of our analytical approach using two datasets suggested that the feature descriptors could efficiently designate a colonic polyp, which subsequently can help the early detection of colorectal carcinoma. Based on the comparison with four deep learning models, we demonstrate that the proposed approach out-performs the existing feature-based methods of colonic polyp identification.

## Introduction

Early detection of disease especially cancer can save millions of lives and substantially reduce the healthcare and economic burden. Numerous techniques have been developed and routinely used for early detection and disease screening, helping our society to reducing the vulnerability of disease at a large extent^1,2^. One such technique is colonoscopy, which has become increasingly popular for early detection and prevention of colorectal carcinoma. Colonic polyps occur frequently and are more lethal than other types. ‘Polyp’ generally describes a mass of tissue over-grown into the lumen of the gastrointestinal tract^3^. The World Health Organization has recommended a thorough colonoscopy examination every three years for patients having colonic polyps^4^. The current process of colonoscopy examination and abnormality detection is based on subjective analysis of the level of abnormality present in a polyp. For the colonoscopy examination, an endoscope scans the whole colon in real time. When the endoscopists find a polyp, they comprehensively study the characteristics of the polyp and assess the level of its abnormality. If the polyp is normal, it can be simply removed through surgical procedures. Otherwise, biopsy sample is collected from that region for further examination such as pathological assays. Since polyps belonging to different categories have only minimal difference in texture, manual categorization is not recommended. Nevertheless, observation bias may lead to an erroneous diagnosis. In this work, automated feature analysis is performed for the classification of polyps into two classes, i.e., neoplastic (abnormal) and non-neoplastic (normal) polyps.

During the visual examination of the polyps, the physicians check different morphological features including shape (sessile or stalked, irregularity in boundary, size of the region, etc.), surface (e.g., irregularity on the surface, color change) and contour (namely smooth to lobular, nodular, fingerlike, etc.) to classify them into different categories (neoplastic and non-neoplastic). In this work, an efficient automated feature representation scheme is proposed for shape, texture, and color features. Andreu-Perez et al^5^ mentioned the importance of understanding the biological processes, which requires the identification and representation of structure-function relationships. In this work, we mainly focus on quantifying the morphological features with proper guidance of physicians.

Colonoscopy image processing has been an area of active research. Studies cover polyp localization and segmentation^6^, as well as feature analysis for various applications. 3-D reconstruction and polyp segmentation in endoscopic videos are also reported in the literature^7^. There are many studies of the classification of polyps using traditional and deep feature learning based methods. In this work, we propose a feature representation scheme for polyp classification. Our goal is to make it more effective on detecting and classifying polyps.

Fu et al^8^ have worked on a large dataset comprising 365 generated images. They extracted texture features from the first component of the principal component transform, representing both spatial and spectral domains. Sequential forward selection (SFS) and sequential floating forward selection (SFFS) were used to select the input feature vectors for classification using machine learning method support vector machine (SVM). Hafner et al^9^ proposed a color vector field followed by computing the similarity between neighboring pixels to describe local texture properties. The resulting feature descriptor is a compact 1D-histogram, which is subsequently used for classification by k-nearest neighbors (KNN) classifier. Mesejo et al^10^ proposed 2D texture features, 2D color features, and 3D shape features for a three-class polyp classification. For texture features, invariant gabor features and rotational invariant LBP features were used. Shape-DNA and kernel-PCA are used for extracting the 3D shape features. Here, PCA refers to principal component analysis. Wimmer et al^11^ have studied curvelet, contourlet, and shearlet transform features. In reference^12^, invariant Gabor texture descriptors were explored followed by classification using SVM. Engelhardt et al^13^ proposed color-GLCM and color-LBP features for classification using SVM. In reference^14^, Bag of words descriptors with spatial pyramid matching (SPM) is used for polyp classification. Sasmal et al.^15^ also worked on texture features using Gabor filter banks in the initial stage followed by discrete cosine transform (DCT) upon the sub-bands.

Recently, deep learning models have been applied for classification^16–18^. Akbari et al^19^ applied the CNN model and achieved an accuracy of 90.28%. Wang et al^20^ developed a deep learning approach on their own generated dataset and achieved sensitivity of 94.38%. Urban et al.^21^ used VGG16, VGG19, and ResNet50 for their dataset to classify colonic polyps. They claimed to achieve an accuracy of 96% for classifying polyps on a generated dataset of size 8641 collected from 2000 patients. Sebastian et al^22^ tried to classify colon polyps based on the Kudo’s classification schema using VGG16 filter as feature extraction method and achieved an accuracy of 83%. Chen et al.^23^ have also worked on accurate classification of colorectal polyps using DNN and acheived a sensitivity of 96.3%. Ozawa et al^24^ have also applied CNN names as Single Shot Multibox Detector to get sensitivity of 92%. A comprehensive review on application of deep learning in colon cancer is provided by Pacal et al^25^. There are some other recent works on endoscopy image analysis with specific focus on gastric cancer, which has greatly enhanced the field of computer aided detection and diagnosis^26–29^.

In the present work, shape feature is extracted using Generic Fourier Descriptor (GFD), while texture and color components are obtained by NonsSubsampled Contourlet Transform (NSCT) with different filters. Analysis of Variance (ANOVA) is used to evaluate the statistical significance in the contribution of different feature vectors between two classes. In the next step, feature selection is performed by fuzzy entropy-based feature ranking algorithm, resulting in optimized feature set. This feature set is then classified using two popular classifiers: Least Square Support Vector Machine (LSSVM) and Multi-Layer Perceptron (MLP). Finally, classifier selection is performed based on six measures: accuracy, specificity, sensitivity, precision, f-score, and g-mean. Five-fold cross-validation is used for performance evaluation and assessment on over-fitting. Our extensive experiments show that the proposed method outperforms the existing feature-based (conventional) approaches for colonic polyp detection. To evaluate the robustness of the proposed work, we evaluated the proposed approach by using two datasets, a generated dataset and a publicly available dataset. The proposed work is compared with four classical deep learning models.

## Results

### General implementation

**Figure 1:**
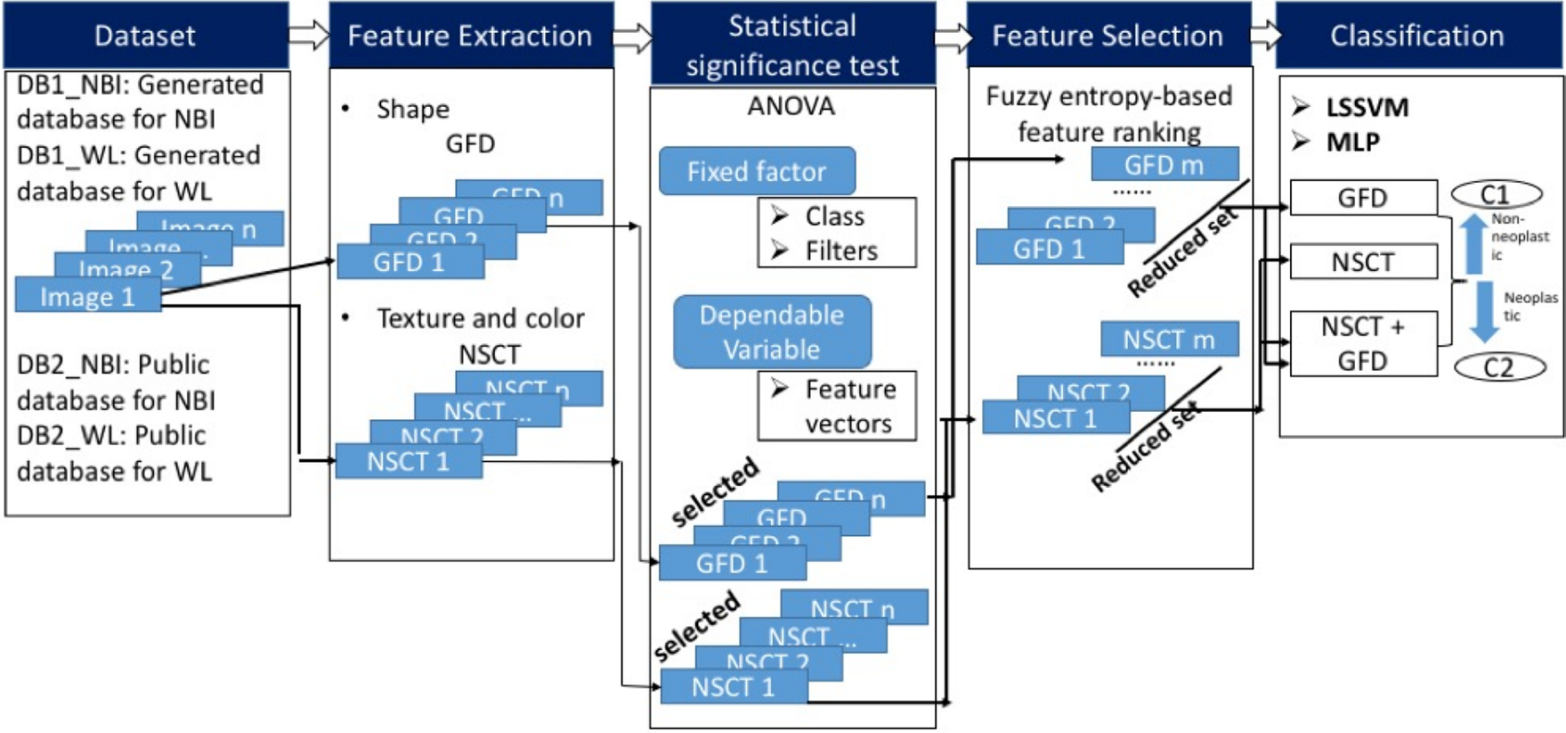
Overview of the proposed work, DB1: Dataset 1, DB2: Dataset 2, C1: Class 1, C2: Class 2.

**Figure 2:**
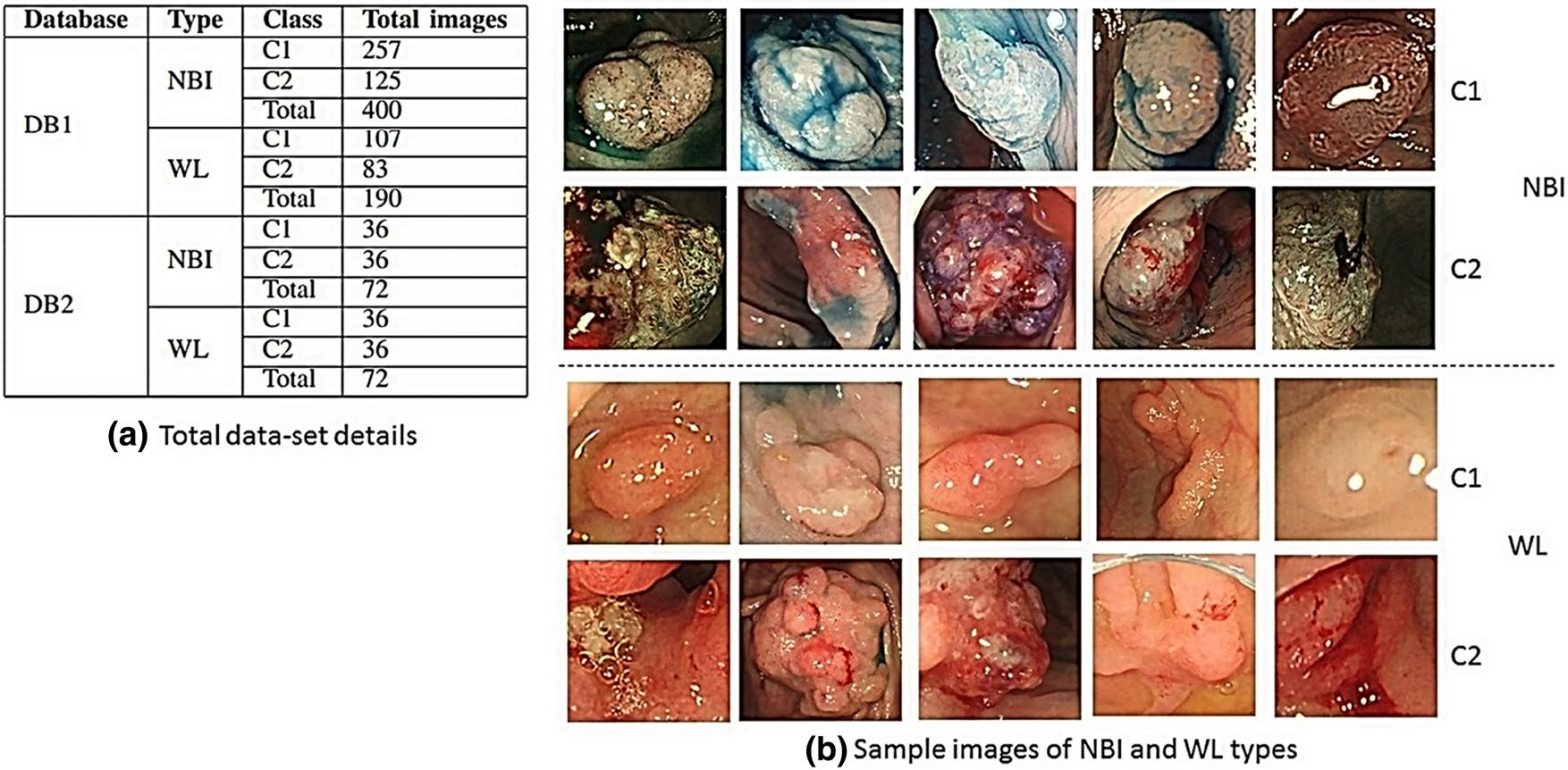
Dataset details with sample images, C1: non-neoplastic samples, C2: neoplastic samples. (**a**) Brief information (viz., type, sample class and total number of images for each sample class) of both the datasets, DB1 and DB2. (**b**) The sample images of NBI and WL types for dataset DB1. Interestingly, these images here are taken from own generated dataset DB1 obtained from Aichi Medical University, Japan [approved by Aichi Medical University ethical committee (January 15, 2018; approval no. 2017-H304)]. Information on the dataset is available in Sub-section ‘Clinical Information’. The layout of the complete figure has been generated in Photo Image Editor Pixelstyle software which is an open source software (URL to download the software: https://apps.apple.com/us/app/photo-image-editor-pixelstyle/id1244649277?mt=12).

All the experiments are performed using MATLAB (The MathWorks, Inc., Natick, Massachusetts, United States) and Lenovo pc (3.20 GHz, Intel Core i5, 4 GB Ram, 64 bit OS). Statistical works are performed using SPSS, version 16 (Statistical Package for the Social Sciences) (Fig. 1 shows the overview of the experimental framework and Fig. 2 shows the dataset used for the study. Details of the same has been included in Materials and Methods section.).

### Results before feature selection

**Figure 3:**
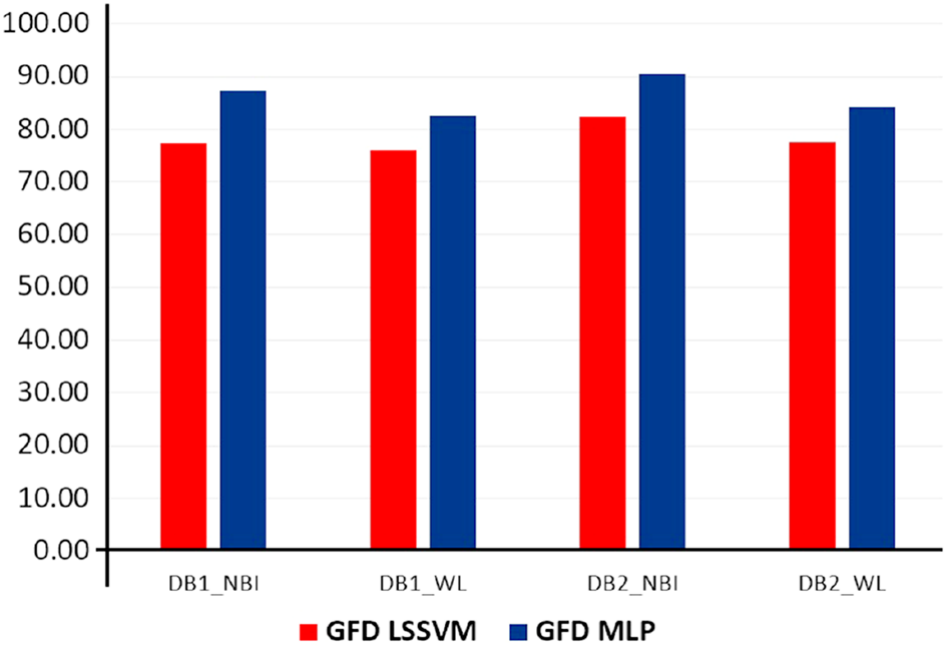
Result of shape features before feature selection.

**Figure 4:**
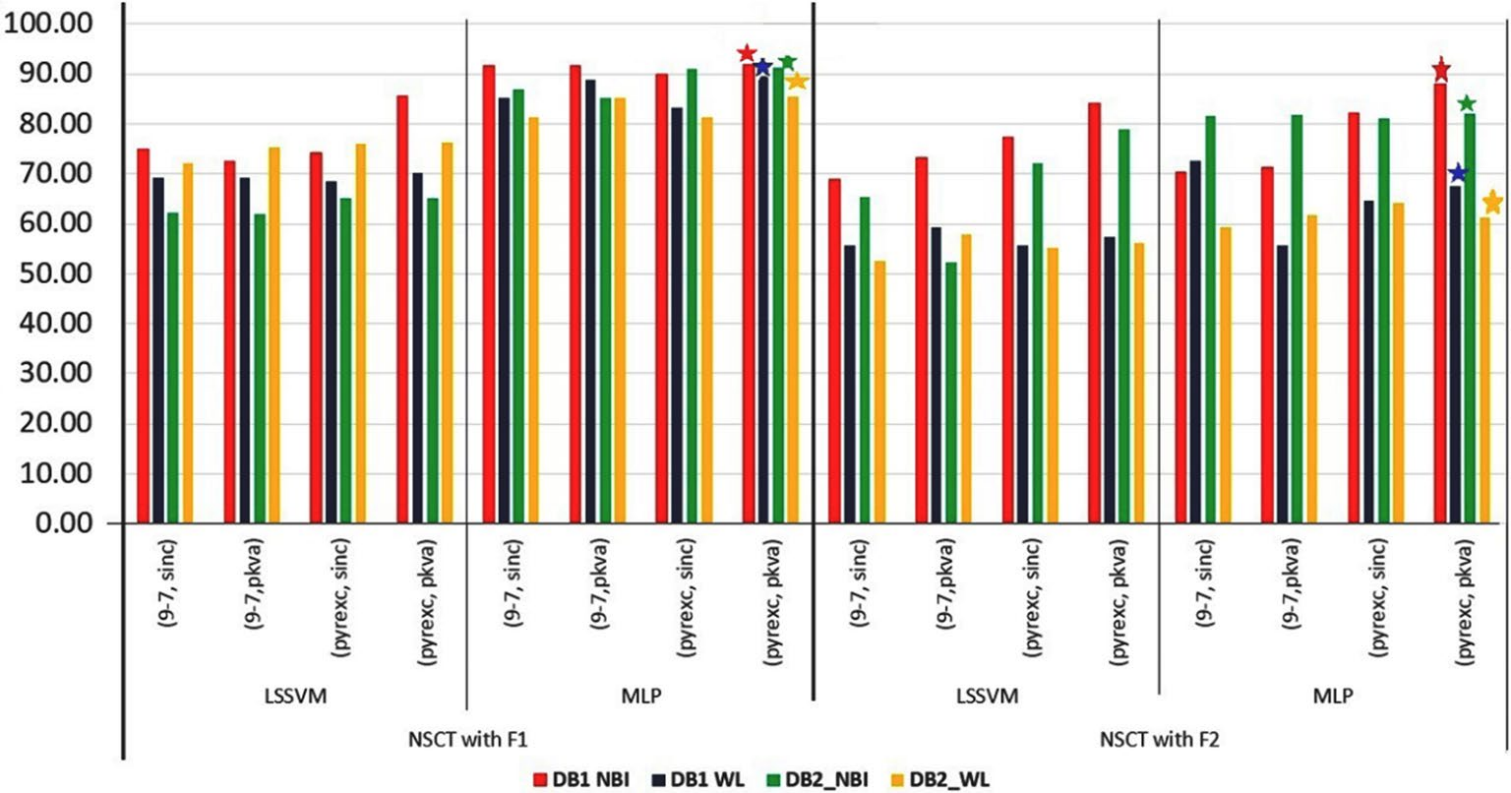
Result of NSCT features before feature selection.

Initially, classifications are performed for all the feature sub-sets for four datasets, viz. $FV_{DB1-{NBI}}$, $FV_{DB1-{WL}}$, $FV_{DB2-{NBI}}$, and $FV_{DB2-{WL}}$, without applying feature optimization. Results are shown separately for shape (Fig. 3) and NSCT (Fig. 4) for proper visualization. Accuracy is used as an assessment measure for two classifiers, namely LSSVM and MLP. For shape features, accuracy for all the datasets (displayed along Y-axis of Fig. 3) are in the range of 75% - 90%. For NSCT, *(pyrexc,pkva)* filter has the best result with accuracy 91.83 % (for $FV_{DB1-{NBI}}$), 88.88% (for $FV_{DB1-{WL}}$), 91.30% (for $FV_{DB2-{NBI}}$) and 85.56% (for $FV_{DB2-{WL}}$) by using MLP. It is observed that ‘*pyrexc*’ NSPF works the best. This filter is derived from 1D using the maximal mapping function with two vanishing moments but exchanging two high pass filters. It can represent smooth edges efficiently. Further, ‘*pkva*’ NSDFB works better than ‘*sinc*’ as it is used to capture the high frequency content of the images like smooth contours and directional edges. It has the best PSNR performance. Due to this property, ‘*pkva*’ performs the best on real colonoscopy images, which has complex features to interpret. Five-fold cross-validation is applied for training and testing the models. For final accuracy, the average accuracy is considered after training and testing for five times. Between feature representation schemes *F*1 and *F*2, *F*1 is giving better accuracy than *F*2. For in-depth analysis of these feature vectors and filters along different classes, the ANOVA test is applied which is in the following section.

### Evaluation of statistical significance test and final feature set design

We intended to analyze whether feature values were affected significantly along with different classes and transform filters for two different feature representation schemes *F*1 and *F*2. To this end, ANOVA was conducted considering the feature values as dependable variables (DVs) and the two factors as fixed factors, namely classes and transform filters (only for NSCT). In a statistical test of a hypothesis, its significance value indicates how the result is different (or better) than that by null hypothesis. A small significance value (*p*< 0.05) indicates the reliable evidence against the null hypothesis. In this case, the two null ($H_0$) and alternate ($H_1$) hypotheses are:

1. $H_{0}^{C}$: (There exists no significance differences of feature values for different Classes) Vs $H_{1}^{C}$:( Feature values differ between classes)
2. $H_{0}^{F}$: (There exist no significance differences of feature values for different filters) Vs $H_{1}^{F}$:( Feature values differ between the filters)

where *C*= Classes, *F*= Features.

The results are shown in Table 1.

**Table 1:** Summary of statistical significance of feature vectors.

	GFD	F1					F2				
		NSCT	DWT	CRT	CVT	RT	NSCT	DWT	CRT	CVT	RT
Class	0.000*	0.000*	0.826	0.671	0.544	0.066	0.000*	0.516	0.072	0.089	0.051
Filters	NA	1	0.59	0.321	0.577	0.59	0.964	0.72	1	1	0.814
**WL**											
Class	0.000*	0.000*	0.606	0.121	0.611	0.077	0.000*	0.061	0.061	0.789	0.022
Filters	NA	0.1	0.725	0.613	0.217	0.1	0.1	0.1	0.121	0.111	0.112

*Less than ‘5% level of significance’.

*Observation based on class as a fixed factor* As shown in Table 1, for feature representation scheme *F*1, class revealed overall influence of DVs to a highly significant level only for GFD and NSCT for both datasets. For feature representation scheme F2, no significant difference is showing along different classes for both datasets. For classification, the factor ‘class’ must reveal overall influence of DVs to a highly significant level. On this basis, we can conclude that only GFD and NSCT can be used for feature extraction. Moreover, feature representation scheme F1 is more efficient than F2 as obtained from the statistical significance test. It can be concluded that mean and variance is not sufficient to represent the transform coefficients.

*Observation based on filters as a fixed factor* There is no significant difference in feature values along different transform filters for NSCT. Thus, we have decided to consider the filter which is giving best accuracy in the classification (from Fig. 4).

The statistical significance of the transforms DWT, CRT, CVT and RT is checked for identifying degree of abnormality. The results showed in Table 1 indicate that they were statistically insignificant and feature values were not affected along with different classes. Hence, we can conclude that NSCT is a better transform than DWT, CRT, CVT, and RT for colonoscopy polyp feature study.

**Figure 5:**
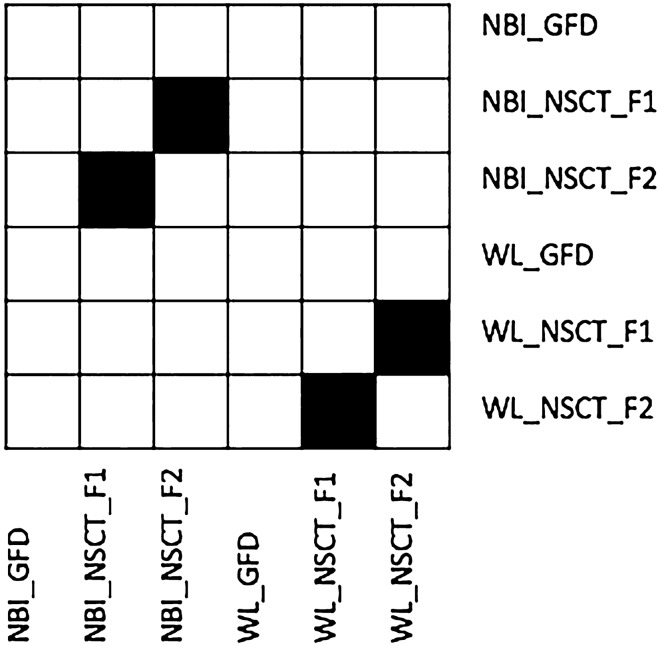
Feature correlation.

*Observation based on correlation* Further, we have applied Pearson’s correlation (*r*) on feature vectors. *r* is a measure of the strength of the association between the two variables. Before considering the final classification, it is desirable to perform experiments on uncorrelated data for obtaining higher performances. Result of r values is displayed in Fig. 5. In this figure, highly correlated values are marked with black cells. The matrix representation will help to understand which feature vectors are highly correlated. As a result, we have found that GFD, and NSCT are not correlated but they are independent. However, $F1_{NSCT}$ and $F2_{NSCT}$ are correlated as expected because they are obtained from the same coefficients, only with different distribution fitting. Hence, we can conclude that GFD, and NSCT are independent with each other, and hence be considered for classification.

### Final feature set design

Based on the statistical test ANOVA, we have selected the following feature sets for *DB*1 and *DB*2.

Shape feature representation using GFD as,

1 $$\begin{aligned} fv(Shape) = f^{GFD} \end{aligned}$$

Texture and color feature representation using NSCT (*pyrexc,pkva*) is as follows. F1 performs better than F2. That is why final feature representation scheme for NSCT is F1.

2 $$\begin{aligned} fv(texture+color) = f^{NSCT}_{(pyrexc,pkva)} \end{aligned}$$

Shape, texture and color features are obtained from the following

3 $$\begin{aligned} fv(shape+texture+color) = f^{GFD} \cup f^{NSCT}_{(pyrexc,pkva)} \end{aligned}$$

Since MLP classifier performance is better than LSSVM, therefore final classifier selected for the study is MLP (based on accuracy as selection measure).

### Feature selection

**Figure 6:**
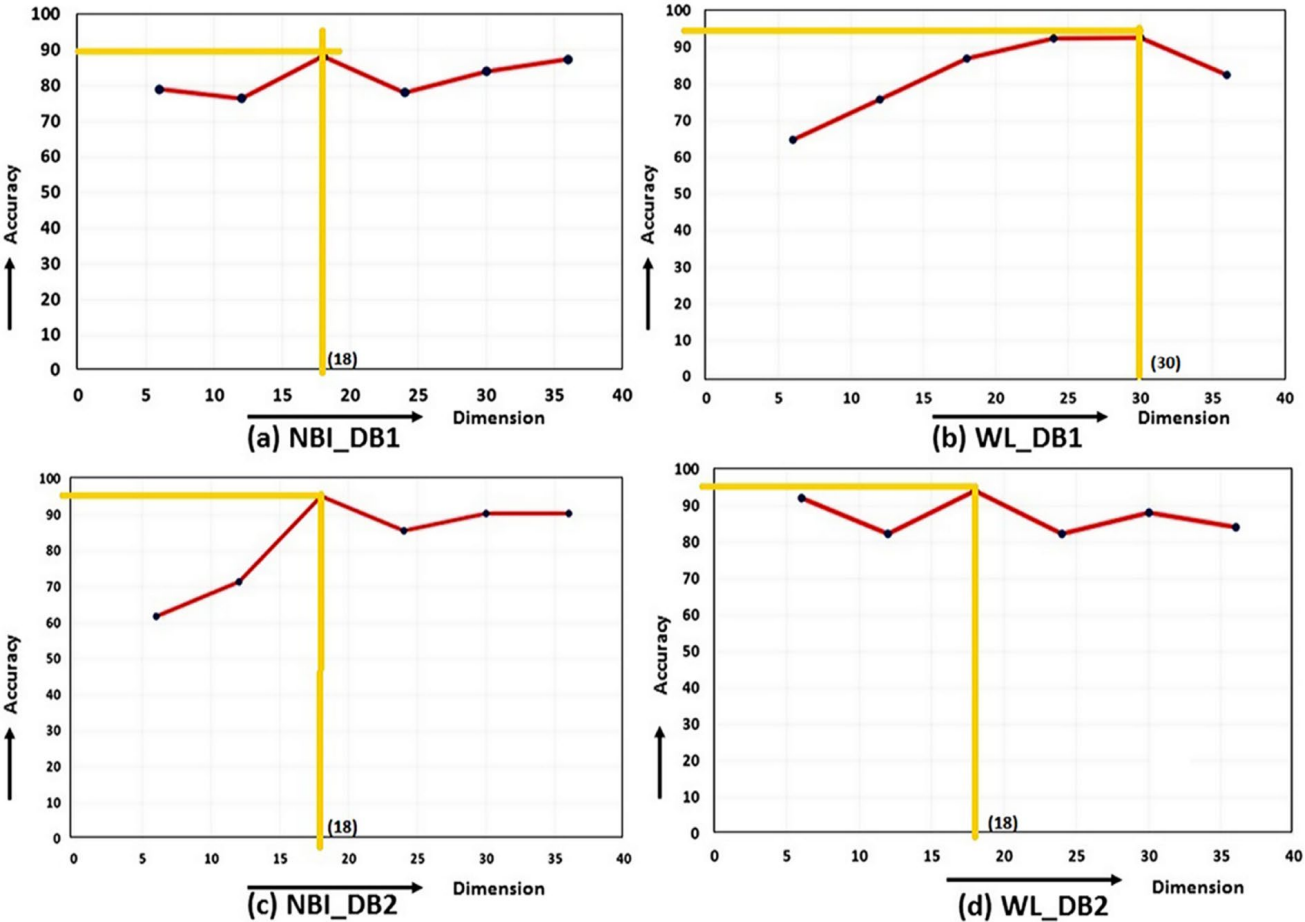
Feature selection in GFD.

**Figure 7:**
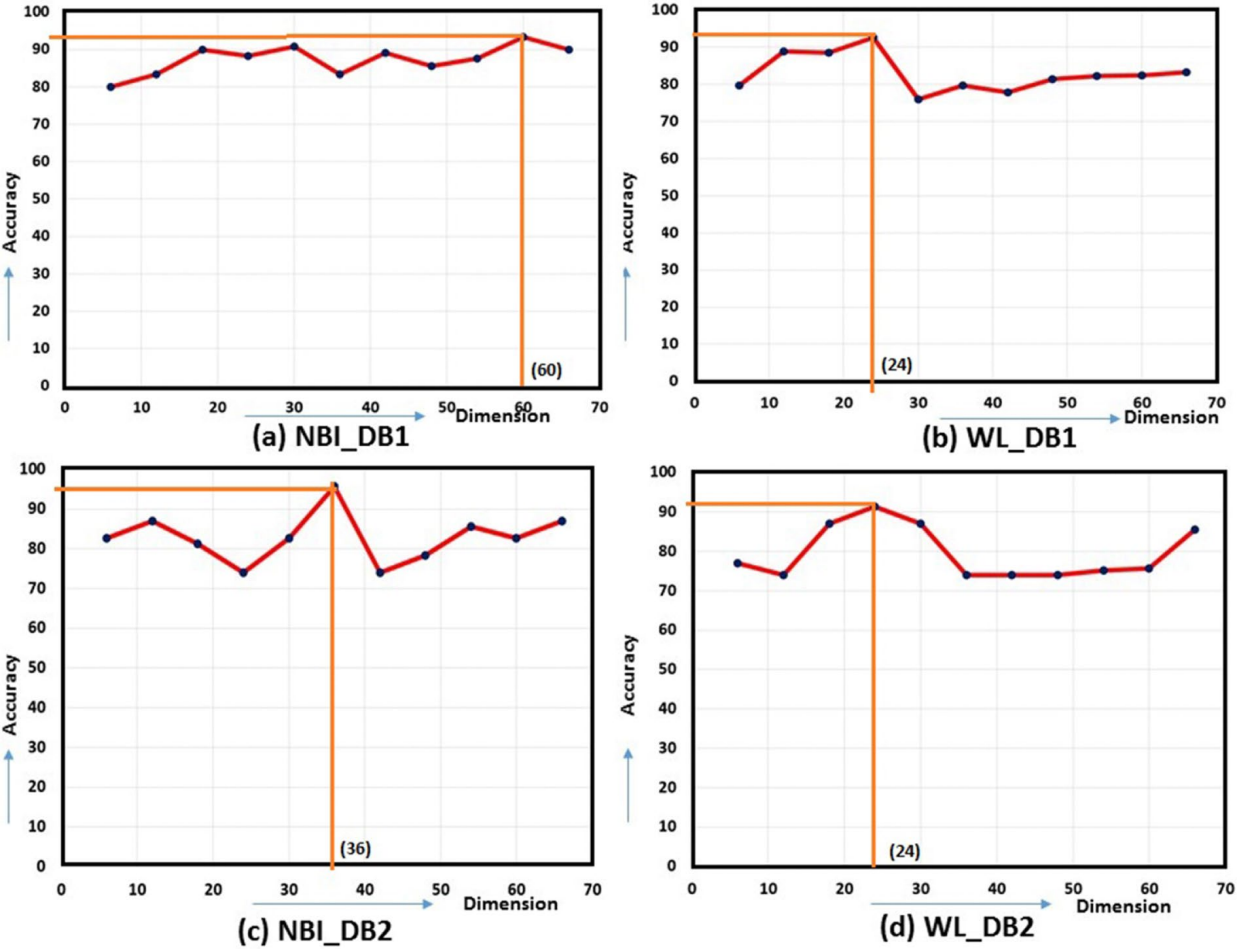
Feature selection in NSCT.

**Figure 8:**
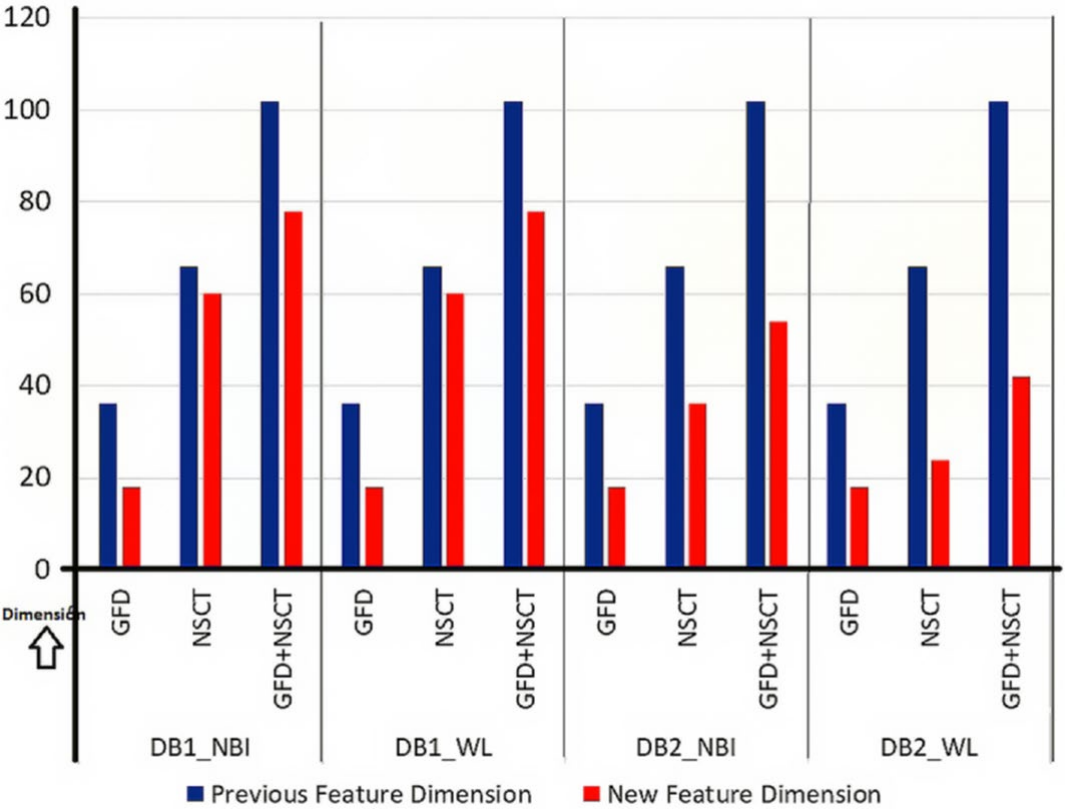
Change in feature dimension.

**Figure 9:**
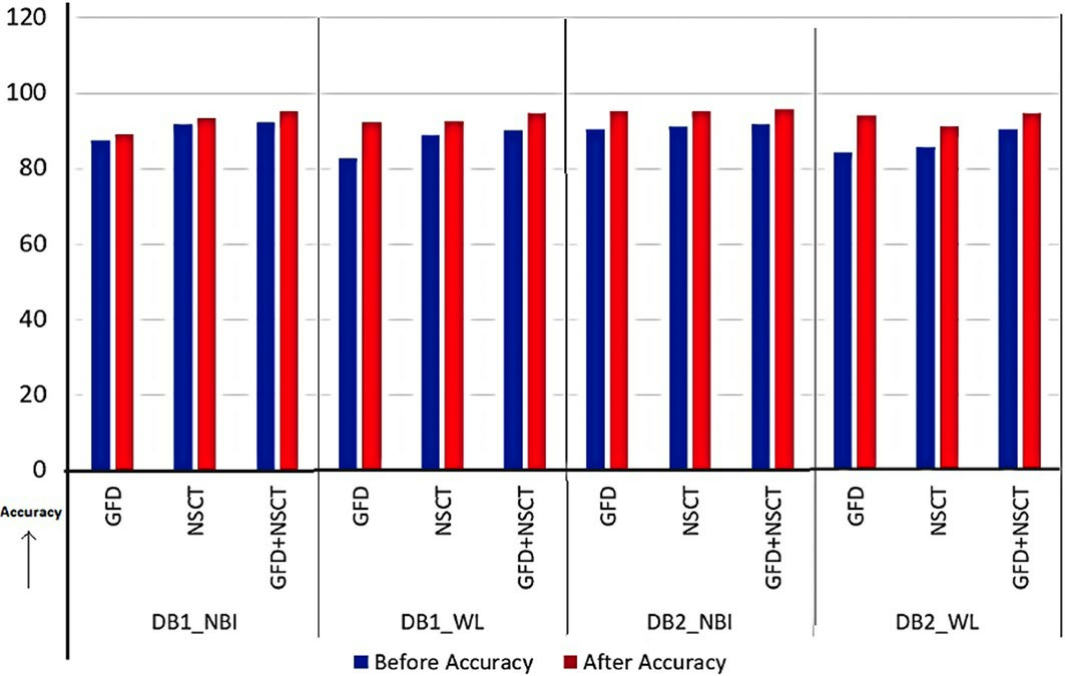
Change in accuracy.

Fuzzy based feature ranking is performed in this work. In doing so, fuzzy entropy based feature selection is applied for ranking the features. Based on the ranking, feature dimensions of size $k=6,12,18,24,\ldots ,m$ have been designed, where $m=36$ for GFD and $m=66$ for NSCT. The minimum feature dimension is considered as 6, because the dimension, as lower than this value would yield low accuracy. Accordingly, classification using MLP is performed for all dimensional features. Finally, peak accuracy is selected, and corresponding dimensions are obtained. Graph of accuracy along different dimensional features are shown in Fig. 6 for shape and Fig. 7 for NSCT. Peak amplitude is displayed in the graphs with its corresponding dimension. There is a significant reduction in feature dimension, which is improving the overall accuracy of the classification. A comparative analysis is displayed in Fig. 8 where we can observe the reduction in feature dimension along four data sets. Change in accuracy using different feature descriptors GFD (for shape), NSCT (for texture and color) and GFD+NSCT (for shape, texture, and color) are shown graphically in Fig. 9. We can observe the increase in accuracy in all the cases, which proves that feature ranking play a major role in removing redundancy. The statistical significance of the final selected feature vectors have been performed using independent sample based t-test or student’s t-test^30^. Here, $\textit{sig} \le 0.01$ value indicates the ‘1% level of significance’ and $\textit{sig} \le 0.05$ indicates ‘5% level of significance’ . The results are displayed in Table 2, where all the significant features are highlighted with the symbool ‘*’.

**Table 2:** Statistical significance test on selected features using fuzzy entropy.

	F	Sig
**DB1_NBI**		
GFD	4.08	0.044197$^*$
NSCT	5.28	0.022133$^*$
NSCT+GDF	5.28	0.022135$^*$
**DB1_WL**		
GFD	12.50	0.003331$^*$
NSCT	13.40	0.000331$^*$
NSCT+GDF	13.42	0.000030$^*$
**DB2_NBI**		
GFD	0.92	0.340670
NSCT	1.22	0.057600
NSCT+GDF	7.56	0.007670$^*$
**DB2_WL**		
GFD	0.92	0.345100
NSCT	2.67	0.108229
NSCT+GDF	8.11	0.006175$^*$

*Significant difference in Independent Sample t-test w.r.t. classes assigned.

### Results after feature selection

We use six measures, namely accuracy, sensitivity, specificity, precision, recall, f-measure, g-mean, to assess performance. All the results along with two different datasets are listed in Table 3. The results are highly satisfactory with GFD+NSCT feature, which is giving highest accuracy of 95.24% (for DB1-NBI) and 94.68% (for DB1-WL). It is also showing consistent performance in the downloaded database with an accuracy of 95.72% (DB2-NBI) and 94.66% (for DB2-WL). Other measures are listed in Table 3. This implies that shape, texture and color are critical in describing the feature of a polyp.

### Comparison with the existing work

For fair comparison, we have selected only those existing works that mainly concentrated on the classification of polyps using specific features. We have selected DB2-NBI datasets for comparison and only two levels of classification (neoplastic and non-neoplastic). Four conventional machine learning methods were compared. The comparative results are listed in Table 4. In Approach 1^10^, Invariant Gabor Texture, Rotational Invariant LBP was used for 2D texture feature analysis, while for 2D color features they have investigated Color naming, Discriminative Color, Hue, Opponent, color GLCM. And 3D shape features were analyzed using Shape-DNA and Kernel- PCA. In Approach 2^12^, Invariant Gabor texture descriptors were classified using SVM, Approach 3^13^ uses color GLCM with *k*NN as classifier. In Approach 4^14^, Bag of Words descriptors with Spatial Pyramid matching were applied. In Table 4, our proposed approach outperforms the four existing ones for colonic polyp classification.

**Table 3:** Results of final classification for three different feature vectors used.

Features	Previous feature vector size	New feature vector size	Avg. accuracy	Avg. sensitivity	Avg. specificity	Avg. precision	Avg. recall	Avg. F-measure	Avg. G-mean
			(±std)	(±std)	(±std)	(±std)	(±std)	(±std)	(±std)
**DB1_NBI**									
GFD	36	18	89.23 (±1.93)	94.05 (±1.43)	82.86 (±1.51)	92.94 (±3.93)	94.05 (±2.03)	93.49 (±1.54)	88.28 (±3.55)
NSCT	66	60	93.33 (±2.64)	93.28 (±3.02)	93.41 (±2.23)	96.26 (±2.42)	94.28 (±3.34)	95.67(±1.99)	93.81 (±3.77)
NSCT+GFD	102	78	95.24 (±0.77)$^*$	95.77 (±0.91)$^*$	91.79 (±1.33)	96.33 (±0.56)	96.77 (±1.11)	96.56 (±0.23)	94.25 (±1.21)
**DB1_WL**									
GFD	36	30	92.22 (±1.66)	92.12 (±2.33)	90.56 (±3.01)	91.65 (±1.22)	96.31(±0.66)	95.14 (±0.91)	90.12 (±0.66)
NSCT	66	24	92.59 (±1.32)	90.12 (±2.66)	92.88 (±3.78)	92.56 (±0.66)	91.76 (±1.04)	89.56 (±1.78)	89.31(±2.76)
NSCT+GFD	102	78	94.68 (±1.12)	95.11 (±0.78)	92.73 (±1.98)	94.01 (±0.82)	90.16 (±2.79)	94.67 (±0.88)	94.33 (±0.45)
**DB2_NBI**									
GFD	36	18	93.13 (±0.55)	90.00 (±1.78)	90.91 (±1.97)	89.89 (±2.11)	90.00 (±1.47)	94.21 (±0.51)	95.28 (±0.22)
NSCT	66	36	95.12 (±0.45)	96.62 (±0.39)	90.00 (±1.23)	94.62 (±0.67)	94.62 (±0.67)	94.62 (±0.86)	92.28 (±0.99)
textbfNSCT+GFD	102	54	95.72 (±0.77)	95.31 (±0.91)	95.00 (±0.20)	93.22 (±1.00)	92.15 (±1.86)	90.45 (±2.91)	94.67 (±0.67)
**DB2_WL**									
GFD	36	18	94.11 (±1.22)	93.45 (±1.59)	92.55 (±1.77)	92.1 (±1.26)	92.1 (±0.29)	89.76 (±2.11)	89.31 (±0.89)
NSCT	66	24	91.30 (±1.91)	90.61 (±2.10)	90.12 (±2.11)	88.76 (±3.19)	89.54 (±3.20)	88.37 (±3.76)	89.31 (±2.77)
NSCT+GFD	102	42	94.66 (±1.46)	92.22 (±1.21)	90.15 (±2.33)	93.76 (±1.67)	94.14 (±1.42)	90.26 (±1.97)	90.66 (±1.55)

**Table 4:** Comparative classification performance of our proposed method in compared to state-of-the-art methods.

	Method	Avg. accuracy	Avg. sensitivity	Avg. specificity	Avg. precision	Avg. recall	Avg. F-Score	Avg. G-mean
	(±std)	(±std)	(±std)	(±std)	(±std)	(±std)	(±std)	(±std)
Proposed	Conventional	**95**.**72** (±**0**.**77**)	95.31 (±0.91)	**95**.**00** (±0.20)	**93**.**22** (±1.00)	92.15 (±1.86)	90.45 (±2.91)	**94**.**67** (±0.67)
Approach 1^10^	Conventional	89.47 (±1.56)*	94.55 (±0.52)	76.19 (±3.21)	91.23 (±1.02)*	94.55 (±1.77)	**92.86 ** (±1.32)*	84.87 (±2.77)*
Approach 2^12^	Conventional	84.21 (±2.69)	90.91 (±1.78)	66.67 (±3.57)	87.72 (±3.18)	90.91 (±1.52)*	89.29 (±2.79)*	77.85 (±2.77)
Approach 3^13^	Conventional	64.47 (±3.97)	74.55 (±4.77)	38.10 (±7.72)	75.93 (±3.77)	74.55 (±5.64)	75.23 (±2.11)	53.29 (±5.42)
Approach 4^14^	Conventional	73.68 (±3.64)	**96**.**18** (±0.22)*	90.52 (±0.57)*	73.97 (±2.91)	**98**.**18** (±0.22)*	84.38 (±0.57)*	30.58 (±5.11)
Approach 5^21^	Deep Learning	92.60 (±1.35)*	–	–	–	–	–	–

*Significant difference in the corresponding evaluation metric between our proposed method and the corresponding method in paired sample t-test.

Bold value indicating the best score of the corresponding evaluation metric (column) among all methods.

We did the second comparison with a deep learning work by Urban et al^21^ (Approach 5 in Table 4). We have tested their architecture on the dataset DB2-NBI and achieved an accuracy of 92.6% with loss of 0.2234, indicating that our proposed work is efficient enough to generate result as deep learning. Note that the running/training time of the proposed algorithm is much lower than the compared approaches. Also, our model have total control on the feature engineering process unlike the deep learning models where features are very difficult to explain, control and interpret.

As a statistical validation, we performed a statistical significance test (paired t-test) of those scores of each evaluation metric (e.g., accuracies, sensitivity, specificity, precision, recall, F-score, and G-mean) individually between our proposed method and each state-of-the-art method in pairwise manner and computed a statistical significance level (*p* value). If *p* value is less than 0.05, the corresponding pairwise comparative result in terms of that specified evaluation metric will be statistically significant (i.e., significantly win of our proposed method over that other method in terms of the specific evaluation metric). In summary, we obtained 11 significant wins among a total of 29 pairwise comparisons in our whole comparison experiments .

The proposed work is compared with four popular deep learning models, namely VGG16^31^, VGG19^31^, ResNet50^32^, and GoogleNet Inception V3^33^. These models are trained and tested with the limited dataset with augmentation. Augmentation method used are cropping, shearing, rotation ($45^{0}, 90^{0}, 120^{0}$) mirroring, skewing (left and right), inverting, and zooming. Different parameters considered are as follows - VGG 16/VGG 19/ResNet 50: batch-size = 32, epoch = 12, ver-bose = 1. For fine tuned version, we have flattened the last layer, and fine-tuned with ReLU activation and adadelta optimizer. For GoogLeNet-V3 fine-tuned model, the hyperparameters in the trained model were: batch-size = 32, number-of-epochs = 12, sgd-learning-rate = 1e–4, momentum = 0.9, transformation-ratio = 0.05. Table 5 displayed the results recorded from the experiments performed on DB2-NBI. Here GoogleNet Inception V3 the hyperparameters in the trained model were: batch-size = 32, number-of-epochs = 12, sgd-learning-rate = 1e–4, momentum = 0.9, and transformation-ratio = 0.05. Here, for GoogleNet Inception V3 shows a satisfactory result with an accuracy of 94.79%. Our proposed method competes well with an accuracy of 95.7%. Although the deep learning model may work well, currently there are not enough large data for training. When sample (feature) size is not large, deep learning models are expected to lose power, leading to low prediction.

**Table 5:** Comparative study of accuracy and loss between our proposed method and state-of-the-art deep learning models.

	Average accuracy	Average loss
Proposed	**95**.**72**	**0**.**1811**
VGG16	82	0.3889
VGG 16 fine tuned	92	0.2054
VGG19	69	0.6894
VGG 19 fine tuned	91.5	0.1823
ResNet50	63	1.2118
ResNet50 fine tuned	68.5	1.1102
GoogleNet Inception V3	90.71	0.3011
GoogleNet Inception V3 fine tuned	94.79	0.2011

Bold value indicating the best score of the corresponding evaluation metric (e.g., accuracy or loss) among all methods.

## Materials and methods

### Clinical information

In the recent years, there are many studies to explore the applications of computer vision in early colonic carcinoma detection. To this end, some researchers have generated own datasets while others have used publicly downloaded datasets. Their objectives are different: some have targeted only on localization of polyps, while others have concentrated on significant frame recognition, classification, among others. Because of these diversities, comparison with the existing works is not convenient. Challenges of existing studies include unavailability of the generated datasets, difficulty in assessing the quality of the images, unavailability of ground truths, among others^10^. Keeping all these challenges in mind, we have performed our experiments on two datasets, one was generated and the other was a public dataset. We attempted to prove that the results obtained from the experiments are consistent between the two datasets. For comparison purpose, we have worked on benchmark of the publicly available dataset only.

Dr. Kunio Kasugai prepared the generated dataset (*DB*1) at the Department of Gastroenterology, Aichi Medical University, Nagakute, Japan, following proper ethical protocols. All methods were carried out in accordance with relevant guidelines and regulations. Prior to colonoscopy examination, the consent of the patients from all subjects has be collected. For generating the dataset, Kasugai has used both Narrow Band Imaging (NBI) and high definition White Light imaging (WL) colonoscopy. Two videos were recorded per patient, one through NBI approach and the other through WL approach. Then he identified the keyframes where the polyp is appropriately visible. Only those significant frames are retained in the dataset and used in the experiments. Video frame extraction and polyp tracking are not considered in this study, as those are entirely different fields of work. Total images included in the study is shown in Fig. 2 along with a sample dataset. Only two classes are included for the study, namely neoplastic and non-neoplastic. This study has been approved by Aichi Medical University ethical committee (January 15, 2018; Approval No. 2017-H304). The collection of data was approved by the institutional review board of Aichi Medical University and the patients were given the opportunity to opt-out. All endoscopic images were de-identified prior to their inclusion in the data set.

The dataset2 (*DB*2) is downloaded from http://www.depeca.uah.es/colonoscopy_dataset/. The details of this dataset are given in Fig. 2a. Both the datasets are composed of frames taken from colonoscopy videos captured using two modalities, namely Narrow-band Imaging (NBI) and White Light (WL). Ground truths of all the images are prepared and verified by the experts.

### Overview of the analytical approach

The overview of the proposed work is displayed in Fig. 1. This work has five major phases as follows.

1. *Phase 1 - Dataset generation:* A dataset has been generated during this phase with two types of images, namely NBI (Narrow Band Imaging) and WL (White Light), and with two groups of images in each type, i.e. non-neoplastic and neoplastic.
2. *Phase 2 - Feature extraction:* Shape, text and color features are extracted. Shape feature is extracted using GFD. Texture and color features are extracted using NSCT with different combination of filters.
3. *Phase 3 - Statistical significance test:* ANOVA is performed to examine whether there is any significant difference among feature vectors along multiple classes and filters. Finally, only significant feature descriptors are selected for final analysis.
4. *Phase 4 - Feature selection and final feature set design:* Fuzzy entropy based feature ranking algorithm is used for feature optimization.
5. *Phase 5 - Classification:* Classification is performed using LSSVM and MLP. Assessments are performed using six measures. The final classes will give the degree of colorectal carcinoma namely non-neoplastic and neoplastic.

A brief description on the methods applied is included in the following sub-sections.

### Shape descriptor GFD

During polyp identification and classification, physicians always prioritize shape features among all others. Major factors include size of the polyp region, irregularity in the polyp, thickness, etc. Some good descriptors are always recommended in the digital domain to quantify the shape features. Shape descriptors mainly divided into two broad categories, namely contour-based and shape-based. Contour-based shape descriptor only concentrates on boundary information avoiding all shape interiors. However, region-based descriptors provide shape information based on all the pixels within a region, which makes it more favorable than the contour-based. In many publications, moments are found to represent the region-based shape descriptors. For two examples, Hu et al^34^ proposed two-dimensional moment invariant and Kim et al^35^ presented a descriptor which is invariant to rotation, scale, and translation. More works can be found in References^36–38^. Zernike moment is considered to be one such strong shape descriptor^37^, but it is unable to capture spectral features in the radial direction and does not allow multi-resolution analysis along with those directions. Note that, GFD is proved to be the best among all without redundant features and allows multi-resolution feature analysis along both radial and angular direction. This concept motivates us to use GFD as shape descriptor. In this work, GFD is extracted from the spectral domain by applying 2-D Fourier transform on polar raster sampled image. This work is motivated by the work of Zhang et al^39^ which proved it to be better than MPEG 7.

For an image *f*(*m*, *n*) , Modified Polar Fourier transform can be obtained as follows:

4 $$\begin{aligned} MPFT(\varrho ,\psi )= \sum _{r}\sum _{j}f(r,\theta _{i})exp[j2\pi (\frac{r}{R}\varrho +\frac{2\pi i}{A}\psi )] \end{aligned}$$

$MPFT(\varrho ,\psi )$ signifies the coefficients along $\varrho$ radial frequency and $\psi$ angular frequency respectively. In Eq. (1), $0 \le r = [(m-m_{d})^{2}+ (n-n_{d})^{2}]^{1/2}$ and $\theta _{i}=i(2\pi /T)(0\le i\le T)$; $(m_{d}, n_{d})$ represent center of mass of the shape; *R* and *A* represents radial and angular resolutions respectively. Here $0 \le \varrho < R$ and $0 \le \psi <A$.

**Figure 10:**
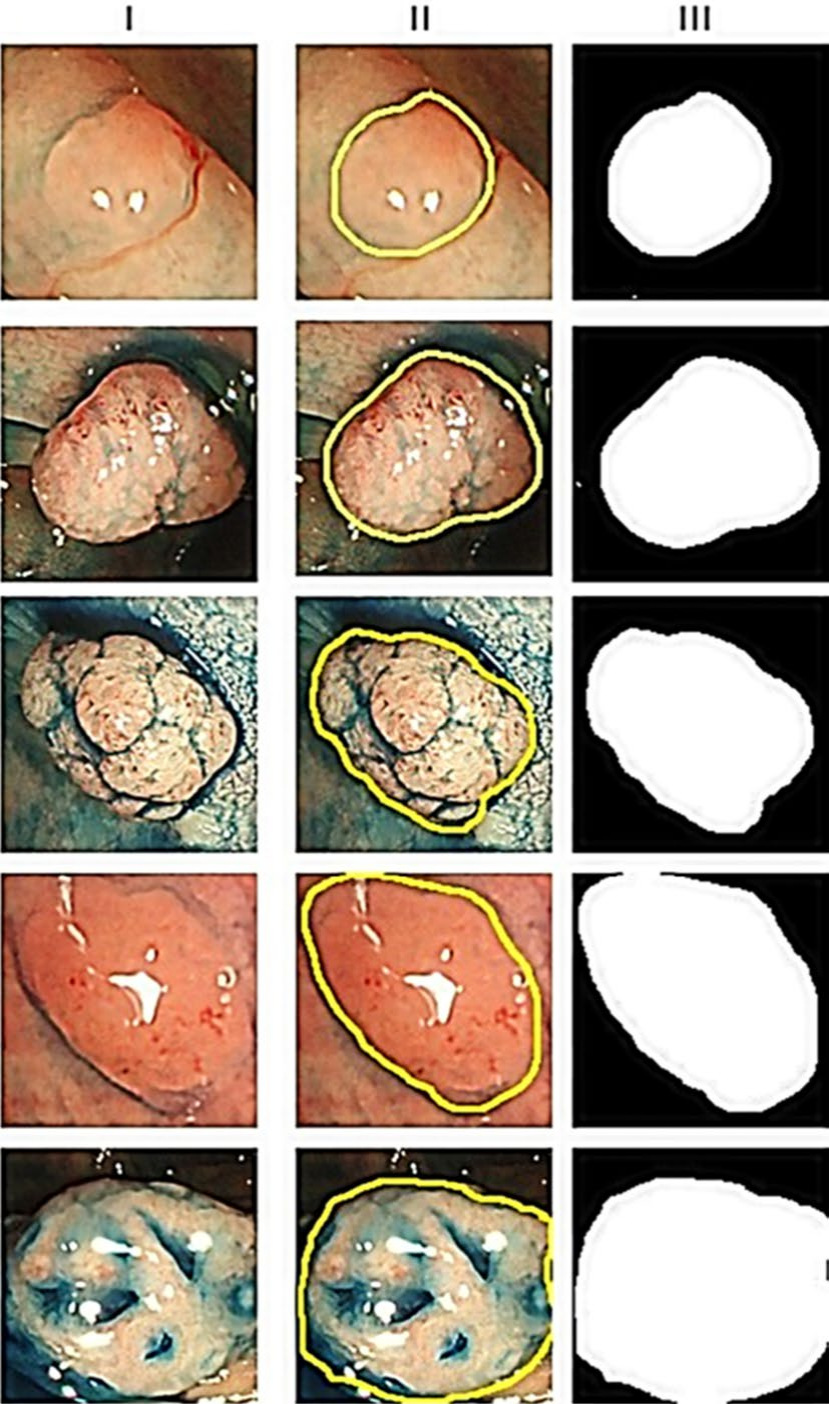
Generation of binary image for shape feature extraction. Column I, II, and III show the original image, ground truth image, and binary segmented image, respectively. All images displayed here are taken from own generated dataset DB1 collected from Aichi Medical University, Japan [approved by Aichi Medical University ethical committee (January 15, 2018; Approval No. 2017-H304)]. The layout of the complete figure has been generated in Photo Image Editor Pixelstyle software which is an open source software (download the software: https://apps.apple.com/us/app/photo-image-editor-pixelstyle/id1244649277?mt=12).

This is followed by normalization to achieve rotation and scale invariance -

5 $$\begin{aligned} \begin{aligned} GFD = \frac{|MPFT(0,0)|}{area}, \frac{|MPFT(0,1)|}{PF(0,0)},\ldots ,\frac{|MPFT(0,x)|}{PF(0,0)}, \ldots \frac{|MPFT(y,0)|}{PF(0,0)}\ldots \frac{|MPFT(x,y)|}{PF(0,0)} \end{aligned} \end{aligned}$$

where *area* denotes the area of the bounding circle of the polyp; *x* and *y* represent the maximum radial and angular frequencies considered, respectively. Since shape features are captured by lower frequencies, first 36 features are considered for the study with 4 radial and 9 angular frequencies. Therefore, final shape descriptor is:

6 $$\begin{aligned} f^{GFD} = GFD (I) , \quad |f^{GFD}|= 36 \end{aligned}$$

where I is the binary segmented image and polyp is the region of interest. ‘ || ’ indicates the dimension of the feature vector. For 2D shape feature, a single frame is considered where the region of interest is clearly visible. This frame is selected by experienced doctors and also ground truth marking of that image is performed. Then, segmentation is performed on the ground truth image using K-mean algorithm. Figure 10 displays how the binary image I is prepared for shape feature extraction.

### Texture and color descriptor

Texture and color descriptors help in quantifying the color changes and irregularity on the surface of the polyp. Multi-resolution transforms are chosen for texture and color extraction as they have the capability of representing images with fewer coefficients, which can be observed along with multiple scales, directions, and resolutions. They are localized in both time and frequency domain, whereas no prior segmentation technique is needed for analysis. Multi-resolution study presents a new direction for image representation on its solid mathematical base. During recent years, much effort has been made in designing directional representation of images such as Ridgelet^40^, Curvelet (CVT)^41,42^, Contourlets (CRT)^43^, NSCT^44,45^, Ripplet (RT)^46^ and Shearlet (SH)^47^ (and many more) under the concept of Multi-scale Geometric Analysis. Wimmer et al^11^ have extracted the features using curvelet, contourlet, and shearlet transforms in application to colonic polyp detection. In the above mentioned papers, none have attempted to understand the statistical significance of the features, which raises questions on their false positive results. In this work, the statistical relevance of DWT, CRT, and RT features were analysed using ANOVA. The results were considered significant by 5% cutoff in the statistical analysis (Table 1).

**Figure 11:**
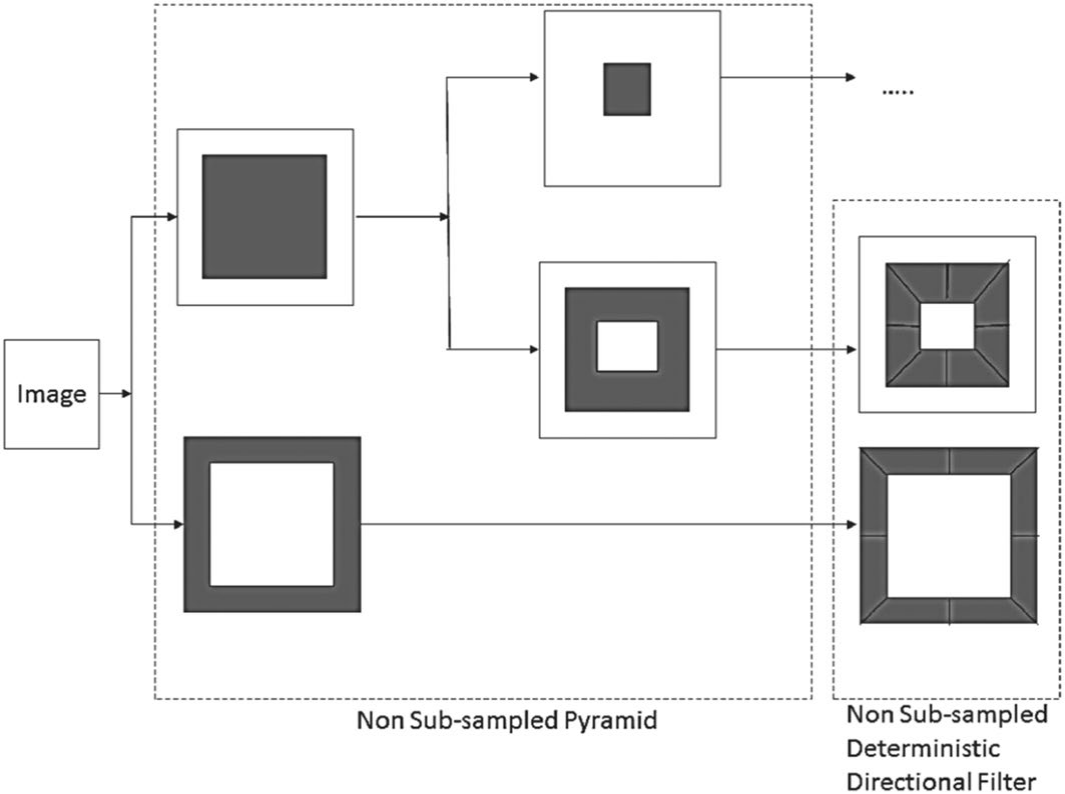
The Nonsubsampled contourlet filter bank. First, the multi-scale decomposition into octave bands by means of the nonsubsampled pyramid decomposition followed by the application of a directional filter bank to the band-pass channels.

NSCT has the property of flexible multi-scale anisotropy, multidimensional expandability, full shift-invariance, and fast implementation^44,48^. It is an improved version of contourlet, where latter is not shift-invariant. The proposed work used the Cunha et al^48^ algorithm to decompose the frequency plane into sub-bands. To this end, Nonsubsampled Pyramidal Filter (NSP) is applied to ensure multi-scale anisotropy and Nonsubsampled Directional Filter Bank (NSDFB) to provide multi-directional expandability. In performing NSCT, firstly an NSP split at each level generates a lowpass version and band- pass version of the image. The latter is obtained from the difference between the image and the prediction. Then, an NSDFB decomposes the high-pass sub-band into several directional sub-bands. The scheme is iterated repeatedly on the lowpass sub-band. A unit of NSCT is shown in Fig. 11. Different combination of pyramidal (pyr) and directional filters (dir) used for NSCT decomposition are $\{(pyr, dir): (9-7, sinc) (9-7, pkva) (pyrexc, sinc) (pyrexc, pkva)\}$. We use NSCT implementation as described in Cunha et al^48^ which is publicly available for coefficient extraction. Before decomposition, the image is converted from RGB to YCbCr where ‘Y’ represents the texture information, and ‘Cb’ and ‘Cr’ represents color information. This is performed to extract the non-uniform characteristic of the image, which is not possible using the RGB model. RGB model is capable of projecting uniform characteristics only. In addition, the ‘Cb’ and ‘Cr’ components are not correlated, which makes it more suitable than other models like HSV.

#### Feature representation for NSCT

Feature descriptors for NSCT are obtained by distribution fitting on the coefficients of each sub-band. Distributions used for this work are Generalized Gaussian Distribution (GGD) and Normal Distribution (ND). Then parameters obtained after the fitting are considered as feature descriptors.

*NSCT coefficients generation* Let us consider a function shown in following equation to explain the transform setup:

7 $$\begin{aligned} \phi = NSCT (I, `pyr\text ', `dir\text ', decom\_ level); \end{aligned}$$

The Eq. (7) decomposes the image *I* using ‘*pyr*’ pyramidal filter and ‘*dir*’ directional filter with decomposition level as $`decom\_level$’, $\phi$ is the transform coefficients obtained. The decomposition level considered for NSCT is [1, 2, 2]. This indicates that there is three pyramidal decompositions and the number of directional decomposition in each pyramid level from coarse to fine resolution is 1, 2, 4, and 4, respectively. The output $\phi$ has cell vectors such that -

1. $\phi _1=[\phi [1] ]$ indicates low pass sub-band image.
2. $\phi _2=[\phi [2,1] , \phi [2,2]]$ are high pass sub-band.(Obtained from 2nd level decomposition)
3. $\phi _3=[\phi [3,1] , \phi [3,2],\phi [3,3], \phi [3,4]]$ are four directional sub-band of the next finer pyramidal level.
4. $\phi _4=[\phi [4,1] , \phi [4,2],\phi [4,3], \phi [4,4]]$ are four directional sub-band of the finest pyramidal level.

$\phi _3$ and $\phi _4$ are obtained from third level decomposition. Therefore, total sub-bands generated is $11(=1+2+4+4)$. The three-color planes of YCbCr results in 33 sets of transform coefficients. From each set of 33 coefficients, two feature descriptors are extracted (using two different method F1 and F2), which leads to a final feature vector of dimension 66.

*F1* GGD based feature representation. In this feature representation, the feature of an image *I* can be represented as follows

8 $$\begin{aligned} \begin{aligned} F_{1}(NSCT) = \lbrace f_{\alpha }^{\phi ^{Y}_{j}}, f_{\beta }^{\phi ^{Y}_{j}}, f_{\alpha }^{\phi ^{Cb}_{j}}, f_{\beta }^{\phi ^{Cb}_{j}}, f_{\alpha }^{\phi ^{Cr}_{j}}, f_{\beta }^{\phi ^{Cr}_{j}} \rbrace \end{aligned} \end{aligned}$$

where $f_{\alpha }^{\phi ^{Y}_{j}}$ represents the alpha parameter of the transform coefficients of *j*th sub-band using *Y* channel, and $f_{\beta }^{\phi ^{Y}_{j}}$ represents the beta parameter of the transform coefficients of *j*th sub-band. Similarly, we have to extract parameters for the other two-color spaces. GGD is used in modeling when the concentration of values around both the mean and the tail behavior is of particular interest.

Here, $\alpha$ and $\beta$ values are used as feature descriptors. The scale parameter $\alpha$ models the width of the probability distribution function (PDF) peak (standard deviation), while the shape parameter $\beta$ is inversely proportional to the decreasing rate of the peak. We apply the Maximum Likelihood method to estimate the parameters $\alpha$ and $\beta$.

*F2* First order statistics based feature representation. In this feature representation, the feature of an image *I* can be represented as follows

9 $$\begin{aligned} \begin{aligned} F_{2}(NSCT) = \lbrace f_{\mu }^{\phi ^{Y}_{j}}, f_{\sigma }^{\phi ^{Y}_{j}}, f_{\mu }^{\phi ^{Cb}_{j}}, f_{\sigma }^{\phi ^{Cb}_{j}}, f_{\mu }^{\phi ^{Cr}_{j}}, f_{\sigma }^{\phi ^{Cr}_{j}} \rbrace , \end{aligned} \end{aligned}$$

where $f_{\mu }^{\phi ^{Y}_{j}}$ represents the mean of the transform coefficients of *j*th sub-band using Y channel, and $f_{\sigma }^{\phi ^{Y}_{j}}$ represents the variance of the transform coefficients of *j*th sub-band. The central limit theorem states that under certain (fairly common) conditions, the sum of many random variables will follow a normal distribution. Every normal distribution is a version of the standard normal distribution whose domain has been stretched by a factor $\sigma$ (the standard deviation) and then translated by $\mu$ (the mean value)

10 $$\begin{aligned} pdf(t;\mu ,\sigma ) = \frac{1}{\sigma }\varphi (\frac{x-\mu }{\sigma }) \end{aligned}$$

where

11 $$\begin{aligned} \varphi (x) = \frac{1}{\sqrt{2\pi }}e^{\frac{1}{2} x^{2}} \end{aligned}$$

$\mu$ and $\sigma$ value obtained after normal distribution fitting will represent $f_{\mu }^{\phi ^{\kappa }_{j}}$ and $f_{\sigma }^{\phi ^{\kappa }_{j}}$ respectively, where $\phi _{i}^{k}$, represents the coefficients of *k* th color channel.

### Final feature sets and database design

Shape, texture and color features are analyzed in this study. Shape feature is extracted using GFD ($f^{GFD}$). Texture and color features are extracted using NSCT ($f^{NSCT}$). For NSCT, four different filters are used which generated the feature vectors $f_{(9/7,sinc)}^{NSCT}$ , $f_{(9/7,pkva)}^{NSCT}$, $f_{(pyrexc,sinc)}^{NSCT}$ and $f_{(pyrexc,pkva)}^{NSCT}$, subscript represents the filter names. For an image *I*, the feature vectors have been generated are as follows

12 $$\begin{aligned} FV = [\lbrace f^{GFD} \rbrace , \lbrace f^{NSCT} \rbrace ] \end{aligned}$$

Where,

13 $$\begin{aligned} \begin{aligned} f^{I}_{NSCT}= [\lbrace f_{(9/7,sinc)}^{NSCT} \rbrace , \lbrace f_{(9/7,pkva)}^{NSCT} \rbrace , \lbrace f_{(pyrexc,sinc)}^{NSCT}\rbrace , \lbrace f_{(pyrexc,pkva)}^{NSCT}\rbrace ,] \end{aligned} \end{aligned}$$

We use two datasets, *DB*1 (generated) and *DB*2 (downloaded) ), each with two types: $DB1-{NBI}, \quad DB2-{NBI}$ (for NBI) and $DB1-{WL}, \quad DB2-{WL}$ (for WL). Therefore final feature vectors generated for the study are - $FV_{DB1-{NBI}}$, $FV_{DB1-{WL}}$, $FV_{DB2-{NBI}}$ and $FV_{DB2-{WL}}$. Size of each dataset is $M \times N$ , where *M* represents total number of images and *N* is the size of feature vectors. M values are available in Fig. 2 and N represents corresponding feature vectors, i.e., 36 for GFD, and 66 for NSCT.

### Feature selection

Fuzzy entropy is an extension of Shannon entropy where the latter is a probabilistic method opposed to fuzzy entropy, which is a possibility-based method. In the fuzzy entropy-based process, fuzzy sets are used in entropy calculations. In this feature selection technique, the required membership of each sample along different feature dimensions is measured. In this work, Khushaba et al^49^ method is used to estimate the membership function. Unlike feature selection techniques like Principal Component Analysis (PCA), Independent component analysis (ICA), etc., it does not fully transform the image features. Rather, it helps in ranking the features based on their contribution to an assigned class. This property helps in efficient monitoring of the features during feature reduction.

Let us consider a feature vector $FV = \lbrace f_{1}, f_{2}, \ldots , f_{l} \rbrace$, where *l* is the number of total features. Fuzzy membership value that *k*th vector will be in *i*th class is -

14 $$\begin{aligned} \mu _{ik} = ( \frac{\Vert f_{i} - f_{k } \Vert _{\sigma }}{r+\epsilon } )^{\frac{2}{\rho -1}} \end{aligned}$$

where $\rho$ is the fuzzification parameter and $\epsilon$ is a value to avoid singularity and $\sigma$ is the standard deviation.

Normalization of the obtained membership values is performed to obtain a standard set of features. In case of total *c* numbers of classes, *c* numbers of fuzzy sets along each feature *f* need to be considered. Each of these reflects the membership degree in *c* problem classes. Fuzzy joint probability of a particular feature vector belonging to a class *c* can be given by the formula

15 $$\begin{aligned} P(f, c_{i}) = \frac{\sum _{k \in U_{i}} \mu _{ik}}{T} \end{aligned}$$

where $P(f,c_{i})$ gives the degree of contribution of a feature to a particular class. $U_{i}$ indicates the indices of the training vector that belong to class *i* and *T* indicates the dimension of feature vector. Joint fuzzy entropy of features of each class can be calculated as follows

16 $$\begin{aligned} H(f, c_{i}) = -P_{f,c} log P_{f,c_{i}} \end{aligned}$$

The complete fuzzy entropy can be obtained from

17 $$\begin{aligned} H(f,C) = \sum _{i=1}^{c} H(f,c_{i}) \end{aligned}$$

Marginal entropy *H*(*f*) can be found as $H(f) = -P_{f_{S_{i}}} log P_{f_{X_{i}}}$ , where $X_{i}$ indicate the *c* numbers of fuzzy sets and $P(f_{X_{i}}$ can be calculated as,

18 $$\begin{aligned} P(f_{X_{i}}) = \frac{\sum _{k} \mu _{ik}}{T} \end{aligned}$$

Marginal class entropy $H(C) = -P_{c_{i}} log P_{c_{i}}$. Then mutual information (MI) between particular feature and class label can be calculated using formula

19 $$\begin{aligned} MI(f;C) = H(f) + H(C) - H(f,C) \end{aligned}$$

In this fuzzy entropy based feature selection method, features are ranked based on increasing and decreasing value of MI based on the application.

### Classification

Two well-known classifiers, LSSVM^44,47,50^ and MLP^51^, are used in this study. These two individual classifiers were selected because of their diversity in performance. All the classifiers were trained and tested individually for the two datasets. The above model is tested and trained with images of the datasets by applying the five-fold cross-validation technique^52^. Main objective of the work is to perform classification^53–55^ where each level signifies the degree of dysplasia in colonic polyps.

## Discussion

In this work, we propose a novel approach to quantify shape, texture, and color features for detecting the stages of dysplasia in polyps. Our work indicates that the shape features to be extracted by GFD, while texture and color features to be extracted by NSCT. ANOVA tests suggest that GFD and NSCT features are significantly different between the two classes (neoplastic and non-neoplastic). Among all the filters of NSCT, we have considered *(pyrexc,pkva)* filters. This is followed by feature optimization using fuzzy entropy based feature ranking algorithm, which could reduce the feature dimensions significantly and also increase the overall accuracy of the classifier MLP. An achieved accuracy of 95.24% in generated dataset and 95.72% in public dataset proved the efficiency of our work. The features obtained are highly efficient in distinguishing polyps irrespective of datasets. We also demonstrate that our method outperforms the four existing ones.

This work only focuses on single frames selected by the doctors. In future, we will integrate it with video tracking and automatic frame selection. The future work also includes the study of 3D features of the polyps. In addition, we can implement our analytical approach into a computational tool for easy use. Furthermore, in future, we will extend this framework with more computational strategies in terms of the analysing of the integrated multi-modal (multi-omics) data of biomedical image and next-generation sequenced array data together for various disease.
